# Primary ciliary dyskinesia: Aetiology, diagnosis and clinical management

**DOI:** 10.1016/j.clinme.2025.100319

**Published:** 2025-04-30

**Authors:** Rachael Collison, Saara A. Hyatali, Antoniya Kamenova, Adam Rashed, Dylan Riley, Kartik Kumar, Janet M. Stowell, Michael R. Loebinger

**Affiliations:** a Department of Respiratory Medicine, Northwick Park Hospital, London North West University Healthcare NHS Trust, London, UK; b Department of Respiratory Medicine, Croydon University Hospital, Croydon Health Services NHS Trust, London, UK; c Department of Respiratory Medicine, St Mary’s Hospital, Imperial College Healthcare NHS Trust, London, UK; d Department of Respiratory Medicine, Ealing Hospital, London North West University Healthcare NHS Trust, London, UK; e Department of Cardiology, Harefield Hospital, Guy’s and St Thomas’ NHS Foundation Trust, London, UK; f Department of Respiratory Medicine, Hammersmith Hospital, Imperial College Healthcare NHS Trust, London, UK; g National Heart and Lung Institute, Imperial College London, London, UK; h Host Defence Unit, Department of Respiratory Medicine, Royal Brompton Hospital, Guy’s and St Thomas’ NHS Foundation Trust, London, UK

## Abstract

Primary ciliary dyskinesia (PCD) is a rare genetic disorder characterised by abnormal function of motile cilia. The condition usually manifests in early life with neonatal distress, chronic sinopulmonary disease and organ laterality disorders. In adults, it is an underdiagnosed cause of bronchiectasis as well as subfertility. This review provides an overview of PCD for clinicians. We discuss its aetiology, its presentation, how it is diagnosed and its multidisciplinary clinical management.

## Highlights

• Recent improvements in genetic testing have shown a greatly increased prevalence of PCD, suggesting a significant cohort of undiagnosed patients.
• PCD should be considered in individuals with a history neonatal respiratory distress, a chronic productive cough, chronic rhinosinusitis and organ laterality defects.
• Diagnosis of PCD is contingent on identifying specific clinical symptoms and investigations such as nasal nitric oxide, ciliary microscopy and genetic testing.
• Clinical management should focus on airway clearance and robust treatment of acute infections.

## Background

Primary ciliary dyskinesia (PCD) is an inherited disorder characterised by abnormal function of motile cilia. It is a genetically heterogenous condition, with inheritance most often following an autosomal recessive pattern. Over 50 genes and more than 2,000 mutations have been identified as causative. The global prevalence has recently been estimated to be as high as 1/7,500, while other studies have previously suggested that its prevalence may be between 1/15,000 to 1/30,000 live births.^1^^,^^2^ Prevalence varies among ethnic groups and may be higher in groups practising consanguinity.^3^ Genetic mutations affect coding of the proteins that form the structure of motile cilia that are found in the upper and lower respiratory tract, reproductive tract and ventricles within the central nervous system.^4^^,^^5^ Estimated to account for more than 30% of cases, the first identified mutations in PCD were *DNAI1* on chromosome 9 and *DNAH5* on chromosome 5, which encode components of the outer dynein arm that powers ciliary beating.^5^

Motile cilia in the respiratory tract comprise microtubules in a “9+2” configuration, in which nine pairs of microtubules are arranged peripherally around two single microtubules. In PCD, dysfunction in the ciliary beating pattern and frequency results in mucus stasis and impaired mucociliary clearance, resulting in a microenvironment of chronic inflammation that is liable to infection. Phenotypic variation may occur with different mutations, leading to heterogenous disease outcomes among patient groups.^6^ Around 70% of PCD cases can be identified with genetic testing of known pathogenic variants, but in up to 30% of cases exhibiting typical clinical symptoms, a known genetic cause is not identified.^7^

### Clinical presentation

The median age of PCD diagnosis is 5 years.^8^ Some patients however remain undiagnosed into adulthood and it remains an underdiagnosed cause of bronchiectasis.^9^ Older age at the time of PCD diagnosis is associated with an impairment in baseline forced expiratory volume in 1 second (FEV1) and greater colonisation with *Pseudomonas aeruginosa*.^10^

**Fig. 1:**
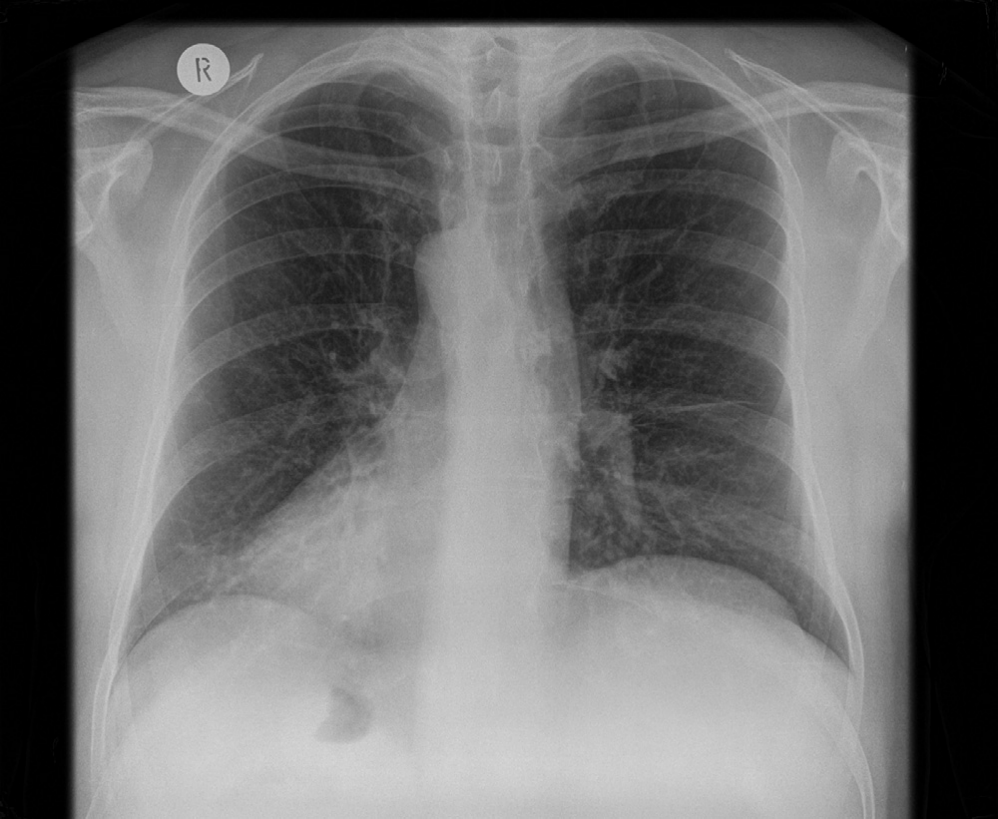
A chest radiograph showing dextrocardia with situs invertus totalis in an individual with PCD.

**Fig. 2:**
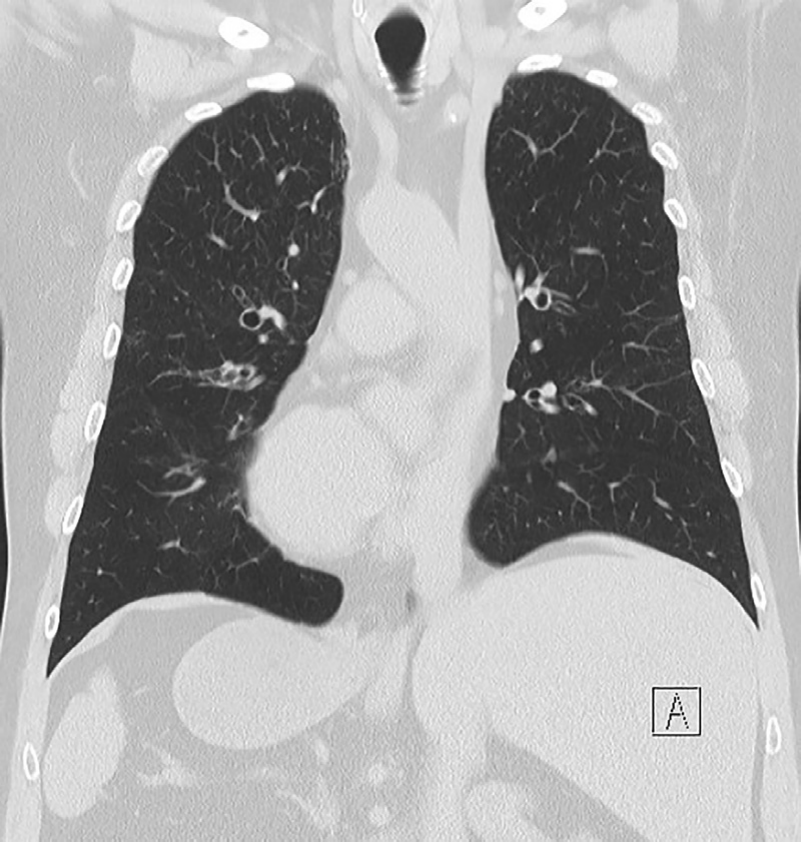
A coronal section of a high-resolution chest CT scan demonstrating bronchiectasis and dextrocardia with situs invertus totalis in an individual with PCD.

PCD should be considered as a potential underlying diagnosis in individuals who report unexplained neonatal respiratory distress, a chronic productive cough (which may have started in infancy), chronic rhinosinusitis and organ laterality defects. Approximately 80% of patients with PCD will have experienced unexplained neonatal respiratory distress requiring supplementary oxygen or respiratory support. The cough can be productive from a young age. Both the cough and rhinosinusitis are typically persistent after prolonged courses of antibiotics. Around half of adults with PCD require sinus surgery. Situs abnormalities are present in around 50% of patients and occur due to the role of nodal cilia during embryological development. These cilia determine left to right organ patterning; if defective, they can result in random laterality of organs. This can lead to a variety of anatomical abnormalities, including dextrocardia or situs inversus totalis (Fig. 1, Fig. 2).^11^ Congenital heart disease and/or situs abnormalities other than situs inversus totalis affect 25% of people with PCD.^11^

Other symptoms associated with PCD include recurrent otitis media, with many patients requiring intervention such as tympanostomy during childhood. Men with PCD are often affected by infertility due to motility issues in the sperm tail, which has a similar structure to cilia. Women with PCD may have reduced fertility, likely due to defective fallopian tube and endometrial cilia affecting the movement of the egg through the fallopian tubes and uterus.

**Fig. 3:**
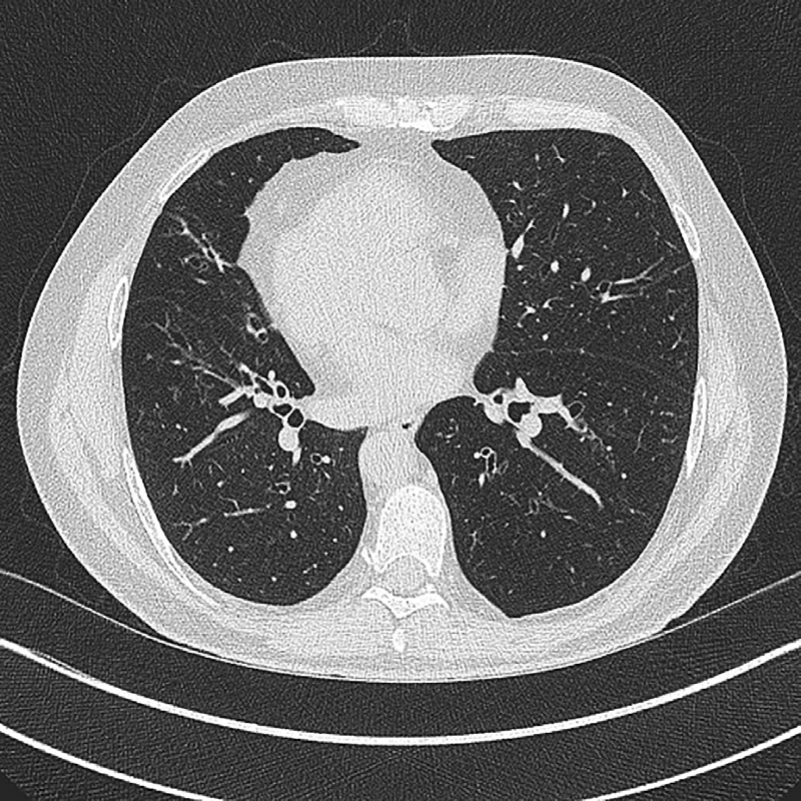
An axial section of a high-resolution chest CT scan demonstrating bronchiectasis and dextrocardia in an individual with PCD.

The most common reason for an acute hospital admission for a patient with PCD is recurrent lower respiratory tract infections. By adulthood, almost all patients will have bronchiectasis, often affecting the middle and lower lobes (Fig. 3).^12^ The lungs may become colonised with respiratory pathogens. *Haemophilus influenzae* and *Moraxella catarrhalis* are among the most commonly cultured bacteria initially. As the pulmonary microbiome changes over time, approximately one third of adults will become colonised with *Pseudomonas aeruginosa*.^13^ PCD is also a risk factor for chronic pulmonary infection with non-tuberculous mycobacteria.^14^

The sinopulmonary manifestations of PCD may resemble those of cystic fibrosis (CF), making sweat chloride testing essential for children under evaluation for PCD. Hereditary immunodeficiencies may also present with recurrent upper and lower respiratory tract infections.

### Diagnosis

There is no single gold standard test for confirming PCD. Diagnostic methods have evolved from early screening tools such as the saccharin test to advanced techniques including genotyping, immunofluorescence staining of ciliary proteins and electron microscopy tomography. Diagnosis typically relies on a combination of clinical findings and specialised tests, including nasal nitric oxide (NO) measurement, high-speed video microscopy analysis (HVMA) and transmission electron microscopy (TEM).^15^

NO is produced in the airways by three nitric oxide synthase (NOS) isoenzymes: endothelial NOS, inducible NOS and neuronal NOS. It is generated in a range of respiratory tract cells, including airway epithelial cells, endothelial cells, neutrophils, mast cells, smooth muscle cells and nerve cells.^16^ NO has been shown to increase the frequency of ciliary beating and to confer a protective effect against infection. Nasal NO levels are low in PCD and the mechanisms underlying this remain to be elucidated.^2^ Measuring nasal NO levels provides a rapid and non-invasive means of screening for PCD. More than 90% of PCD patients present with low nasal NO levels when using a specific cutoff value (<77 nL/min). False positives can occur in CF or viral infections.^17^

HVMA and TEM can be used to assess ciliary beat pattern/frequency and ciliary structure respectively. Challenges associated with HVMA include difficulties in assessing ciliary beat abnormalities if the cilia have been damaged by chronic inflammation. Assessment of the ciliary beat after regeneration of the cells by cell culture is a useful technique.^18^

Next-generation sequencing is increasingly used as a first-line diagnostic tool for PCD. The most frequently identified affected genes include *DNAH5, DNAH11, CCDC40, DNAI1, CCDC39* and *SPAG1*.^19^ Current genetic panels fail to provide a definitive diagnosis in up to 30% of cases. This gap is expected to narrow as new PCD-related genes are incorporated into testing.^20^

### Management

There are currently four PCD management centres in the UK: Royal Brompton Hospital in London, Southampton General Hospital in Southampton, the Central England PCD Management Service in Leicester and Birmingham, and the North of England PCD Service in Leeds and Bradford. The first three units also provide the national PCD diagnostic service. All patients in whom PCD is suspected should be referred to a specialist centre for diagnosis and further management if the diagnosis is confirmed. A multidisciplinary approach is central to the management of patients with PCD. As PCD can be a complex disease affecting multiple systems, many healthcare professionals may be involved in patient care, including respiratory physicians with expertise in PCD, cardiologists with expertise in congenital heart disease, ENT surgeons, audiologists, clinical geneticists, dietitians, respiratory physiotherapists, clinical nurse specialists and psychologists. Many of the principles underlying effective PCD management overlap with those that apply to non-CF bronchiectasis.^21^

Airway clearance is a crucial part of management in PCD due to the lack of normal mucociliary movement. Patients should be taught personalised airway clearance techniques by a respiratory physiotherapist with expertise in PCD. If mucus expectoration is challenging, mucoactive therapies such as hypertonic saline nebulisers and carbocisteine may be offered, although generally sputum in PCD is less dehydrated than in CF.^22^ Cough clearance can be effective when augmented with handheld positive expiratory pressure devices.^23^ Preventative measures such as vaccinations against influenza, COVID-19 and pneumonia are of paramount importance. Additionally, some patients with laterality defects may be functionally asplenic and require accelerated vaccinations.^24^

For sinonasal disease and recurrent otitis media, regular review by an ENT specialist is required. Patients with chronic rhinosinusitis disease should be taught nasal sinus lavage. Sinus aplasia and hypoplasia is common and patients may benefit from functional endoscopic sinus surgery, even though the prevalence of nasal polyposis in patients with PCD is lower compared to patients with CF.^25^ Regular audiology checks are necessary to avoid missing hearing impairment, which may be conductive or sensorineural due to either recurrent otitis media or due to antibiotic ototoxicity. Some patients may require speech therapy, while others may need hearing aids. If patients are contending with subfertility, they may benefit from advanced reproductive techniques.^26^

Compared to bronchiectasis of other aetiologies, PCD has been associated with lower quality of life scores in relation to treatment burden and social functioning.^27^ Tailored disease-modifying therapies for patients with PCD are in their early phases. A recent clinical trial has demonstrated a small improvement in FEV1 after 4 weeks of combination treatment with idrevloride (a selective epithelium sodium channel inhibitor) and hypertonic saline.^28^ Pre-clinical trials are currently underway to test mRNA therapy for correction of specific genetic defects.^29^

## Summary

PCD is an underdiagnosed cause of adult bronchiectasis. Maintaining a high index of clinical suspicion is vital for timely diagnosis. Maintaining effective airways and sinonasal clearance is the mainstay of longterm management. Acute infections require prompt antibiotic treatment with extended regimens as guided by culture results. A multidisciplinary approach to clinical management is required.

**Key take home points**

1) Recent improvements in genetic testing have shown a greatly increased prevalence of PCD, suggesting a significant cohort of undiagnosed patients.
2) PCD should be considered in patients with neonatal respiratory distress, a chronic productive cough, chronic rhinosinusitis and laterality defects.
3) Diagnosis of PCD is contingent on identifying specific clinical symptoms and investigations such as nasal nitric oxide, ciliary microscopy and genetic testing.
4) Clinical management should focus on airway clearance and robust treatment of acute infections.

## Consent for publication

The patient provided written consent.

## Funding

This article did not receive any specific grant from funding agencies in the public, commercial, or not-for-profit sectors.

## CRediT authorship contribution statement

**Rachael Collison:** Conceptualization, Writing – original draft. **Saara A. Hyatali:** Conceptualization, Writing – original draft. **Antoniya Kamenova:** Conceptualization, Writing – original draft. **Adam Rashed:** Conceptualization, Writing – original draft. **Dylan Riley:** Visualization, Writing – review & editing. **Kartik Kumar:** Conceptualization, Writing – review & editing. **Janet M. Stowell:** Supervision, Writing – review & editing. **Michael R. Loebinger:** Supervision, Writing – review & editing.

## Declaration of competing interests

The authors declare the following financial interests/personal relationships which may be considered as potential competing interests:

Co-author is an associate editor and editorial board member of Clinical Medicine - KK. Co-author declares consulting fees from Armata, 30T, Astra Zeneca, Parion, Insmed, Chiesi, Zambon, Electromed, Recode, Boehringer Ingelheim, Ethris, Mannkind, AN2 Therapeutics; and payment or honoraria for lectures, presentations, speakers bureaus, manuscript writing or educational events from Insmed - MRL. If there are other authors, they declare that they have no known competing financial interests or personal relationships that could have appeared to influence the work reported in this paper.
